# Supplementary figures and images for: Characterisation of the human uterine microbiome in non-pregnant women through deep sequencing of the V1-2 region of the 16S rRNA gene
Source: PeerJ. 2016 Jan 19;4:e1602. doi: 10.7717/peerj.1602 (PMC4730988; doi:10.7717/peerj.1602)

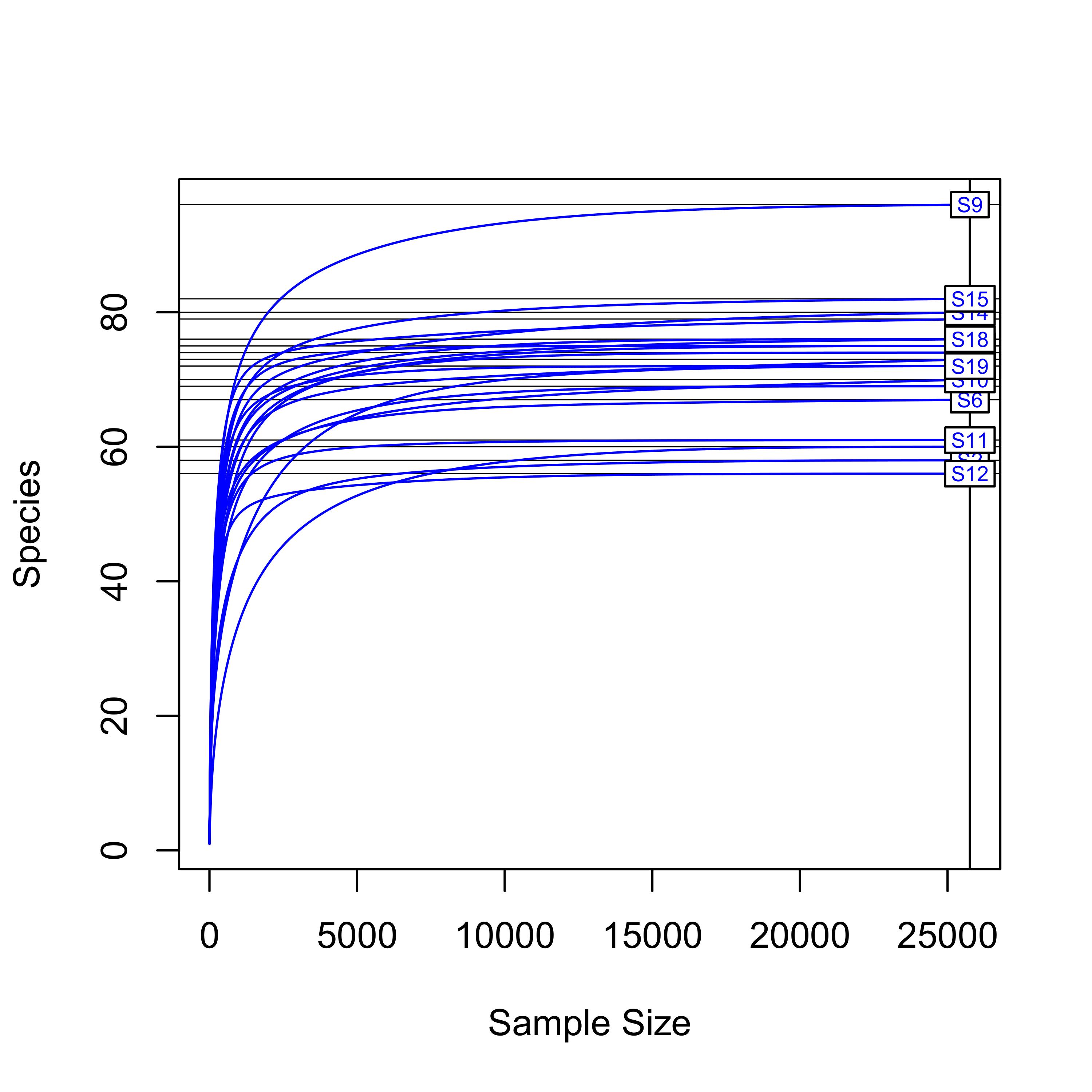

Supplement: Figure S1 [file peerj-04-1602-s001.jpg]
